# Ethnobotany: A Living Science for Alleviating Human Suffering

**DOI:** 10.1155/2016/9641692

**Published:** 2016-07-17

**Authors:** Rahmatullah Qureshi, Shahina A. Ghazanfar, Hassan Obied, Viliana Vasileva, Mohammad A. Tariq

**Affiliations:** ^1^Pir Mehr Ali Shah Arid Agriculture University, Rawalpindi 46000, Pakistan; ^2^Royal Botanic Gardens Kew, Richmond, Surry TW9 3AB, UK; ^3^School of Biomedical Sciences, Charles Sturt University, Wagga Wagga, NSW 2678, Australia; ^4^Institute of Forage Crops, “Gen. Vl. Vazov” Street 89, 5800 Pleven, Bulgaria; ^5^California Department of Food and Agriculture, Sacramento, CA 95814, USA

Since time immemorial, plants served as the first source of medicine to treat ailments. Man learnt about the therapeutic use of plants through trials and errors. This knowledge has been orally passed from generation to generation which led to the development of the traditional health care system, practiced in various countries of the world [1]. Ethnobotanical studies discover plant resources that can be used for targeting novel compounds leading to the development of new medicaments for treating especially complicated and minor diseases [2]. Today, ethnobotany and ethnopharmacognosy are being used for targeting new compound. Due to being rich in diversity, tropical regions may play key role in providing germplasm with new leads [1].

It is estimated that 80% of the world's population lives in developing countries and over 80% of the world's population rely on plant-derived medicines for their primary health care needs [3]. Based on the personal experience, people knew therapeutic potential of the medicinal plants without rationale of their efficacy. Because of advancement, we have a better understanding of the healing powers of plants due to presence of multifunctional chemical entities for treating complicated health conditions.

The ethnobotany provided significant information that led to isolation of active compounds from the recent past like morphine from opium, cocaine, codeine, digitoxin, and quinine [4–6]. It is worthwhile to mention that a dozen of effective valuable drugs are discovered during the last 40 years from higher plants. The very common ones are diosgenin derived from* Dioscorea deltoidea*; reserpine from* Rauwolfia serpentina*; pilocarpine from* Pilocarpus* spp.; vincristine/vinblastine from* Catharanthus roseus*; digoxin/digitoxin from* Digitalis* species [7]; arteether (trade name Artemotil), a recent antimalarial drug is obtained from artemisinin-a sesquiterpene lactone isolated from* Artemisia annua* [8]; galantamine (also known as galanthamine, trade name Reminyl) isolated from* Galanthus woronowii* [9, 10].

Drug discovery from plant lore and traditional medicines are reemerging. Ethnobotanical studies exposed various medicinal plants for discovering miraculous drugs which are still available in the market. Even today, various areas of the world have a unique tradition of plant lore for alleviating human suffering as well as their domesticated animals. There is a need to document such valuable information before it is permanently lost. Based on such data, new medicaments can be predicted through undergoing experimentation which may be of potential use to treat various complicated human diseases. The plant kingdom is an implicit gold mine of new chemical compounds which are still waiting to be explored. It is estimated that there are approximately 500,000 to 750,000 species of higher plants existing on earth and less than 10% of them are examined for their biochemical constituents [11].

Keeping the importance of ethnobotany, an interdisciplinary field of study, this special issue was dedicated to the integration of past and present use of plants reporting traditional/folk medicinal use along with latest development for validation of such information through scientific studies. This special issue is a collection of seven articles portraying the use of medicinal plants and their therapeutic potential. The issue is mainly divided into two main themes; the first one describes the traditional knowledge of plants and the other one describes validation of such knowledge through* in vitro* assays.

From ethnobotanical perspective, four articles are selected. K. C. Chinsembu carried out an ethnobotanical study from Livingstone, Southern Province, Zambia. He reported 94 medicinal plant species which are used to treat HIV/AIDS-related diseases. He stressed to confirm the antimicrobial efficacies, pharmacological parameters, cytotoxicity, and active chemical ingredients of the discovered plants. In a study carried out by M. Meragiaw et al. reported ethnobotanical enumeration of Delanta (Ethiopia) to examine the use of medicinal plants and impacts of the 1984/85 resettlement program on the local people's knowledge on herbal medicine and its uses. They reported 133 species belonged to 116 genera and 57 families in treating 76 human and livestock ailments. Their analysis showed that the resettlement program has both positive and negative impacts on nature rehabilitation and local knowledge along with many human induced threats. S. F. Sabran et al. discovered ethnomedical knowledge of plants used for the treatment of tuberculosis by the Jakun community of Kampung Peta (Malaysia). They identified 23 plants which are used by the community for the same purpose.* Dipterocarpus sublamellatus* was recorded for the first time as novel species to treat tuberculosis. They urged that findings of this study are worth being further investigated for conservation strategies and are worthy of verifying their ethnomedical claims scientifically. A survey was conducted by M. A. Agbor and S. Naidoo to document ethnomedicinal use of plants by the traditional healers in treating oral health problems in Cameroon. They reported 52 plants which are being used for the management of toothache, sore throat, mouth sores, abscess, broken tooth and jaw, tooth sensitivity, mouth thrush, dental caries, gingivitis, sinusitis, tonsillitis, xerostomia, oral syphilis, oral cancer, TMJ pain, halitosis, tooth bleaching, and dental extraction.

From the bioactivity assessment point of view, four articles were selected. M. K. Swamy et al. investigated the effect of different solvents on the extraction of phytoconstituents of* Lantana camara* leaves and their antioxidant and antibacterial activities. They reported that the methanol solvent yielded the highest phenolic (92.8 mg GAE/g) and flavonoid (26.5 mg RE/g) content revealing antioxidant activity. Methanol extract had the highest inhibition activity against all the tested microbes. They identified major compounds such as hexadecanoic acid (5.197%), phytol (4.528%), caryophyllene oxide (4.605%), and 9,12,15-octadecatrienoic acid, methyl ester, (Z,Z,Z)- (3.751%) through GC-MS. A laboratory study carried out by N. Jayawardena et al. investigated antioxidant and starch hydrolase inhibitory activities of 10 spices through* in vitro* model of digestion mimicking the gastric and duodenal conditions. The total phenolic contents in all spice extracts had significantly increased following both gastric and duodenal digestion revealing a correlation with the antioxidant assays quantifying the water-soluble antioxidant capacity of the extracts. They concluded that the tested spices had a significant source of total phenolics, antioxidant, and starch hydrolase inhibitory activities. Finally, S. Baral et al. studied* in vivo* ameliorating effect of myrrh (AEM) on scopolamine-induced memory impairments using mice model. The AEM was estimated with (2E,5E)-6-hydroxy-2,6-dimethylhepta-2,4-dienal as a representative constituent through HPLC. The oral administration of AEM ameliorated the scopolamine-induced memory impairments and increased the phosphorylation of Akt and ERK in the hippocampus of mice brain.

We anticipate that this special issue will provide traditional knowledge of plants existing in various traditional communities to manage and treat various diseases as well as their scientific validation through bioassay assessment.
